# Childcare Correlates of Physical Activity, Sedentary Behavior, and Adiposity in Preschool Children: A Cross-Sectional Analysis of the SPLASHY Study

**DOI:** 10.1155/2018/9157194

**Published:** 2018-11-11

**Authors:** Amar Arhab, Nadine Messerli-Bürgy, Tanja H. Kakebeeke, Stefano Lanzi, Kerstin Stülb, Annina E. Zysset, Claudia S. Leeger-Aschmann, Einat A. Schmutz, Andrea H. Meyer, Simone Munsch, Susi Kriemler, Oskar G. Jenni, Jardena J. Puder

**Affiliations:** ^1^Obstetric Service, Lausanne University Hospital, Lausanne, Switzerland; ^2^Department of Clinical Psychology and Psychotherapy, University of Fribourg, Fribourg, Switzerland; ^3^Child Development Center, University Children's Hospital Zurich, Zurich, Switzerland; ^4^Children's Research Center, University Children's Hospital Zurich, Zurich, Switzerland; ^5^Epidemiology, Biostatistics and Prevention Institute, University of Zurich, Zurich, Switzerland; ^6^Department of Psychology, University of Basel, Basel, Switzerland

## Abstract

**Background:**

The childcare (CC) environment can influence young children's physical activity (PA), sedentary behavior (SB), and adiposity. The aim of the study was to identify a broad range of CC correlates of PA, SB, and adiposity in a large sample of preschoolers.

**Methods:**

476 preschool children (mean age 3.9 yrs; 47% girls) participated in the Swiss Preschoolers' Health Study (SPLASHY). PA and SB were measured by accelerometry. Outcome measures included total PA (TPA), moderate-to-vigorous PA (MVPA), SB, body mass index (BMI), and skinfold thickness (SF). PA measures consisted of both daily PA during CC attendance days and overall daily PA (CC and non-CC days).

**Results:**

We identified the following CC correlates for higher TPA and/or higher MVPA or lower SB during CC attendance days: older age, sex (boys), more frequent child-initiated interactions during CC, mixing different ages within a group, and the presence of a written PA policy in the CC (all *p* ≤ 0.02). The CC correlates for overall TPA and/or MVPA or lower overall SB including both CC and non-CC days were the following: older age, sex (boys), more frequent child-initiated interactions during CC, mixing different ages within a group, less parental PA involvement in the CC, and having a larger surface area in CC (all *p* ≤ 0.046). Correlates for lower SF were sex (boys) and parental PA involvement in the CC (all *p* ≤ 0.02), and, for lower BMI, only increased age (*p*=0.001) was a correlate.

**Conclusions:**

More frequent child-initiated interactions and mixing different ages in CC, the presence of a written PA policy, and a larger CC surface are correlates of PA and SB during CC attendance days and/or of overall PA. Parental involvement in CC PA projects was a correlate for reduced body fat. These novel factors are mostly modifiable and can be tackled/addressed in future interventions.

## 1. Introduction

The recent World Health Organisation (WHO) report about childhood obesity prevention measures includes the promotion of physical activity (PA) and the reduction of sedentary behavior (SB) during the early years [1]. Since many young children spend a large portion of their week in childcare (CC) [2, 3], the CC is an ideal context within which to target children's health behaviors. Indeed, research has shown that interventions in CC can have an impact on young children's activity behaviors and/or on their body weight [3, 4], but there is still a need for more data [5]. Therefore, it is important to expand our knowledge on CC correlates of young children's PA, SB, and body weight in order to tailor future interventions [5, 6].

Childcare correlates of PA and SB differ by country and CC [6–8], and most studies have focused on PA-specific correlates. Initial studies that identified CC correlates originated from the US showed that active opportunities, staff PA training and education, portable play equipment, and sufficient outdoor space were positively related to children's in-care PA behavior [9–11]. However, these US studies have not looked at correlates of SB. On the other hand, a Canadian study found that sedentary environment, sedentary opportunities, and fixed play equipment were correlates of in-care SB [12]. Three studies from Australia have identified written PA policy, staff-led structured PA, staff joining in active play, less time spent indoor before going outdoor, use of indoor space for gross motor activities, lower child-staff ratios, presence of outdoor fixed equipment, and increased number of outdoor spaces with natural ground coverings as positive correlates of young children's in-care PA [7, 13, 14]. In regard to correlates of SB, lower child-staff ratio, using of indoor space for gross motor activities, and the presence of outdoor fixed equipment were identified as correlates of less in-care SB [7, 13]. In three European studies, other correlates such as active opportunities in CC, size of indoor area per child, and location of preschool building on the playground have been linked to in-care PA and/or SB in preschool children [15–17]. For example, data from the UK showed that only one correlate, e.g., active opportunities, was associated with children's in-care PA [16]. According to the authors, correlates of the CC environment may have a more limited influence on children's PA in countries such as the UK, where policies advocate child-driven play and moving freely indoors and outdoors [16].

As PA, SB, and obesity are related and are all important health outcomes of children's lives [18], it is important to include not only correlates of PA and SB but also of childhood adiposity. We are aware of one study that evaluated CC correlates of adiposity [19]. The authors found that less minutes spent in SB, more time spent in active play, less offers of high fat/high sugar foods, and less opportunities for sedentary activities during CC attendance days were associated with lower BMI or lower risk of being overweight. Clearly, additional studies investigating CC correlates of obesity in young children are needed.

When investigating CC correlates, the socioecological model provides a useful framework for conceptualizing potential correlates of children's behavior and health across 5 domains: demographic/biological, psychological/cognitive/emotional, behavioral, sociocultural, and physical environment [20–22]. Such a framework will allow us to have a broader and comprehensive perspective across the whole spectrum of the ecological model to clearly identify all potential correlates of PA, SB, and obesity. For example, there is a lack of knowledge about CC correlates at the sociocultural level such as organisation of different age classes or policies of the CC [23]. Additionally, several correlates of PA, SB, and adiposity could also be important novel correlates within the CC setting: socioeconomic status (SES) of the CC [24–26], language region of the CC [27, 28], and psychological and behavioral correlates of the children while in CC, such as peer relationship problems and eating behavior [29–31].

The previously mentioned studies have all evaluated PA correlates by focusing on PA during CC attendance. To our knowledge, only one study examined the effect of the CC environment on children's PA levels over the entire day [32]. The authors found during one single day of observation that 60 minutes of outdoor time or active time during CC attendance was related to increased overall PA of the entire 24-hour day. It could indicate a compensatory effect of CC correlates by either reducing or increasing PA at home. It's important to study if CC correlates also impact on overall daily PA and SB. Thus, it is important to include also days without CC attendance, as it's the overall daily PA behaviors that are related to many important health indicators [33].

The aim of the current study was to investigate CC correlates of (1) PA and SB during CC attendance days, (2) overall daily PA and SB including all days, both CC and non-CC days, and (3) adiposity using data of a large cross-sectional study of 2- to 6-year-old preschoolers. We specifically aimed to include a large range of correlates and integrate more novel correlates based on the socioecological model.

## 2. Methods

### 2.1. Study Sample and Design

The Swiss Preschoolers' Health Study (SPLASHY) is a multisite prospective cohort study including 476 preschool children aged 2 to 6 years within two sociocultural areas of Switzerland (German and French speaking parts) (ISRCTN41045021). Preschool children were recruited from 84 CC within five cantons/provinces of Switzerland (Aargau, Bern, Fribourg, Vaud, and Zurich), which together made up to 50% of the Swiss population in 2013. Recruitment lasted from March 2014 until December 2015. The selection procedure was stratified according to one stratum with 4 levels: urban community (>100,000 inhabitants or biggest city in the canton/province) and rural community, each with high socioeconomic status (SES) and low SES defined according to the median maternal educational level of the community [34, 35]. For the larger urban communities, the list of CC centers was divided into high and low SES using the definition of SES according to the Swiss neighbourhood index of socioeconomic status [25]. The detailed study design and the overall objectives have been published previously [36]. The present analysis focuses on the baseline cross-sectional assessment. The study was approved by all local ethical committees (No. 338/13 for the Ethical Committee of the Canton/Province of Vaud as the main ethical committee). Parents provided written informed consent and children provided oral consent. Data collection was conducted in parallel at all study sites according to standardized procedures.

### 2.2. Assessment of the Outcome Variables

#### 2.2.1. Physical Activity and Sedentary Behavior Assessment

Children's PA and SB were objectively monitored using an accelerometer (wGT3X-BT, ActiGraph, Pensacola, Florida, USA). Children were asked to continuously wear the accelerometer around the right hip for seven consecutive days, including at nights. The device was removed for water-based activities, e.g. showering or swimming. Children, parents, and CC staff received detailed instructions on the use of the activity monitor. PA data (raw data) were collected at a sampling frequency of 30 Hz and downloaded in 3-s epochs. For data analysis, periods of 20 minutes and more of consecutives zero readings were removed and considered as nonwear time [37]. All recordings between 9 pm and 7 am were excluded, as this most likely reflected the hours spent sleeping. Measurements with a minimum of 10 hours recordings were considered a valid day. Epoch length was set at 60 seconds to determine the level of total PA (TPA) as mean accelerometer counts per minute (cpm), moderate-to-vigorous PA (MVPA; min/day), and SB (min/day). MVPA and SB were defined as ≥2120 counts/60-s and ≤239 counts/60-s, respectively, using Butte et al. cutoffs [38], developed specifically for young children using the same accelerometer model. The above age-specific (preschoolers) data collection and processing criteria were based on a recent review [39]. Physical activity measures included daily PA during CC attendance days (full-day attendance) and overall daily PA (including all days, both CC and non-CC days), the latter to evaluate the impact of the correlates on overall PA. For 394 (83%) children, measurements across at least 3 days (2 weekdays and 1 weekend day) were available, whereas for the remaining 82 (17%), only one valid measurement day was available. We therefore tested whether these two groups differed from each other with respect to mean values for any of the eight outcomes used in our study, but there were no significant differences (*p* > 0.05 for any of the eight outcomes, using *t*-tests for independent samples). Therefore, the two groups were collapsed.

#### 2.2.2. Adiposity Assessment

Two different adiposity measures (BMI and skinfold thickness) were assessed in the CC center by 5 different well-trained examiners (CL, AZ, KS, AA, and NM). Height was measured to the nearest 0.1 cm with a stadiometer and weight to the nearest 0.1 kg (Seca, Basel, Switzerland) using standardized procedures. BMI was then calculated as weight/height squared (kg/m^2^). All measurements were taken barefoot and in light clothing. Overweight and obesity were defined based on the WHO [40] criteria. Skinfold thickness (SF) was measured using standard procedures [41] in triplicate to the nearest 0.1 mm with Harpenden calipers (HSK-BI, British Indicators, UK) at the triceps, biceps, subscapular, and suprailiac crest. The sum of all four skinfolds was calculated and referred to as “skinfold thickness or body fat.”

### 2.3. Childcare Correlates

The correlates of the CC environment (Table 1) were mainly assessed through a modified Nutrition and Physical Activity Self-Assessment for Child Care (NAP SACC) Questionnaire [42], completed by the main CC educator responsible for the group of the assessed children. We used the socioecological model of health behavior proposed by Sallis et al. [20] to conceptualize and classify potential correlates according to five domains: (1) demographic/biological, (2) psychological/cognitive/emotional, (3) behavioral, (4) sociocultural, and (5) physical environment [20]. A detailed description of all 35 potential correlates is provided in Table 1. Nutritional correlates (*n*=15) were only used for adiposity as outcome variable.

#### 2.3.1. Demographic and Biological Domains

Five variables from the demographic/biological domain were included in the analysis. Age and sex were extracted from parental report. We also included the sociocultural language region of the CC, SES, and the rural-urban location of the community where the CC was located (see “Study sample and design” for more details).

#### 2.3.2. Psychological, Cognitive, and Emotional Domains

Three variables from the psychological domain were included that assessed educators and children while in CC, as children's social and peer problems are known to be related to childhood obesity [30]. Thus, “child-initiated interaction” (“child goes toward peers”) was examined using the item “good friend” of the “peer relationship problems” scale from the Strengths and Difficulties Questionnaire (SDQ) [43]. It assesses how easily the child goes towards peers. It is thus a child-initiated interaction. “Playing peers” was assessed in order to grasp the number of peers the child plays with during CC [44]. Both items were assessed by the main educator. We also investigated the support of the educators for children by rating the overall “staff support” using direct observation by the PhD and postdocs during the testing. For the latter, we looked at support of educators for children in finding solutions to problems. We thereby used a scale ranging from 1 to 5 with higher numbers signifying more support.

#### 2.3.3. Behavioral Domain

Based on previous research, eating behavior was chosen to assess behavioral correlates [1, 30, 31]. We used three items from the Child Eating Behavior Questionnaire (CEBQ) to investigate the contribution of individual differences in eating behavior (food approach and food avoidant, while in CC) to the development of obesity [45]. Item selected for the “food approach” subscale was child's interest in food (*enjoyment of food* scale). Items selected for the “food avoidant” subscale were child eating when upset (*emotional overeating* scale) and child leaving food on the plate after meals (*satiety responsiveness* scale). The main educator in charge completed the questionnaire.

#### 2.3.4. Sociocultural Domain

Twenty variables from the sociocultural domain were selected. Mixing different ages within a CC group was assessed via CC questionnaire and defined as the number of age classes within a CC group. Five PA items and 13 nutritional items from the NAP SACC were considered representing the global CC policies. For example, daily structured PA, written PA policy, and staff nutrition training were included. Parental PA involvement in the CC was also assessed, as parental involvement is a key element in obesity prevention [4, 46].

#### 2.3.5. Physical Environment Domain

Six variables from the physical environmental domain were selected. CC surface was assessed and represents the ratio of the CC surface (m^2^) per number of children since classroom and playground size are important characteristics in promoting PA in CC settings [9]. Childcare attendance of the child was based on the parental report and represents the number of full days per week children attend the CC. In addition, four PA-related items from the NAP SACC questionnaire were included.

### 2.4. Statistical Analyses

In order to analyse the association between CC environment and children's PA, SB, and adiposity, we used regression model techniques. Children's PA and SB outcomes were as follows: total PA, moderate and vigorous PA, and SB (all 3 both during CC attendance days and overall daily PA). Adiposity outcomes were as follows: BMI and SF. These were each analysed in a separate model.

Two different regression models were applied. First, we used a multiple regression model which included the entire list of putative correlates to enable comparison with previous studies. However, the intraclass correlation coefficient (ICC) between CC was close to 0, and thus we did not use multilevel regression analyses. Because multiple regression models often suffer from overfitting leading to models having low predictive accuracy when predicting new samples [47], we also used a second model, i.e., a variable selection procedure, the least absolute shrinkage and selection operator (lasso) [48]. The lasso model is more biased but exhibits strongly increased predictive accuracy compared to multiple regression models. The lasso technique eliminates unimportant correlates from the model by setting their coefficients to zero while the relevant correlate variables remain in the model, their coefficients being usually shrunk towards zero. Note that the two coefficients for age and sex were deliberately not shrunk and thus always kept in the lasso model, as prior knowledge had demonstrated their influence on PA and adiposity [20, 23, 49]. Predictive accuracy of both the multiple regression model and the lasso were determined by ten-fold cross validation [50]. Performance measures reported were the variance explained (*R* ^2^) and root mean square error (*RMSE*). For the lasso model, we set the tuning parameter alpha to 0.95 [51].

All outcomes and correlates were transformed if necessary to meet regression analysis assumptions (normality and homoscedasticity). For the lasso model, all variables were standardized prior to analysis. Since for the lasso no tests of significance are yet readily available, we refrain from reporting *p* values [52, 53]. To run the lasso model, the two R packages glmnet and caret were used [54, 55].

The data contained missing values. Overall, 14% of values were missing (between 0 and 35%, depending on the variable), and 77% of all children and 84% of all variables had at least one missing value. Therefore, we used multiple imputation techniques to estimate missing values. To this end, prior to any analysis, missing values were repeatedly (i.e., fifty times) imputed using chained equations as implemented in the R package mice [56]. This resulted in fifty complete datasets in which missing values had been replaced by estimated values. Subsequent regression models were thus run fifty times using each of the complete datasets in turn, and results then pooled across the fifty datasets. Since to our knowledge no technique exists to date for combining lasso-based results from several data files, we determined the importance of each potential correlate by computing the mean and standard deviation of lasso coefficients across all fifty data files. In addition, we counted the number of times each putative correlate was contained in the best fitting lasso model according to cross validation (0–50). Note, however, that a value near 50 does not necessarily point to a correlate with high accuracy if the best fitting lasso model had a bad fit. Thus, mean standardized lasso coefficients give a better picture of the importance of a putative correlate. We added the lasso results in the appendix and only refer to major concordances and discordances in the text.

## 3. Results

The descriptive characteristics for all children are summarized in Table 2. Mean age was 3.9 ± 0.7 years and 47% of the participating children were girls. Participants provided an average of 5.6 ± 0.9 days of valid PA data, with a mean wearing time of 12.8 hours ±0.06 per day.

### 3.1. Daily Physical Activity during Childcare Attendance Days (Full-Day Attendance)

Multivariate associations between the 20 potential correlates across all domains and TPA, MVPA, and SB during CC attendance days are presented in Table 3(a). Five variables from the demographic/biological, psychological/cognitive/emotional, and sociocultural domains were identified as correlates of PA: age (older), sex (boys), child-initiated interaction with other children during CC, mixing different ages within a CC group, and the presence of a written PA policy were positively associated with TPA and MVPA during CC attendance days (all *p* ≤ 0.02). These five correlates accounted for 19% and 22% of the variance of time spent in TPA and MVPA, respectively. Three of 20 tested correlates from the psychological/cognitive/emotional and sociocultural domains were inversely related to SB during CC attendance days: child-initiated interaction with other children during CC, mixing different ages within a CC group, and the presence of a written PA policy were associated with less time spent in SB (all *p* ≤ 0.04). These three correlates accounted for 17% of the variance variability of the time spent in SB.

### 3.2. Overall Daily Physical Activity (during All Days, Childcare, and Nonchildcare Days)

Associations between potential correlates across all domains and TPA, MVPA, and SB for overall PA are presented in Table 3(b). Four variables from the demographic/biological, psychological/cognitive/emotional, and sociocultural domains were identified as correlates of overall PA: age (older), sex (boys), child-initiated interaction with other children during CC, and mixing different ages within a CC group were positively associated with TPA and MVPA (all *p* ≤ 0.045). These four correlates accounted for 20% of the variance of the time spent in TPA. For MVPA, CC surface size from the physical environment domain was additionally identified as a correlate (*p*=0.047). Age (older), sex (boys), child-initiated interaction with other children during CC, mixing different ages within a CC group, and CC surface size accounted for 27% of the variance of time spent in MVPA. For overall SB, two correlates from the demographic/biological and psychological/cognitive/emotional domains were associated with less SB: sex (boys) and child-initiated interaction with other children during CC (*p* ≤ 0.04). One correlate from the sociocultural domain was associated with more SB: parental involvement in PA projects of the CC (*p*=0.03). Parental involvement in PA projects of the CC accounted for 14% of the variance of time spent in SB.

#### 3.2.1. Adiposity

Multivariate associations between the 35 potential correlates (including nutritional variables) with BMI and skinfold thickness are presented in Table 4. For BMI, only age was inversely associated with BMI (*p*=0.002). Age accounted for 11% of the variance of children's BMI. For skinfold thickness, sex (boys) and increased parental involvement in PA projects of the CC were associated with lower skinfold thickness (*p* ≤ 0.02). Sex (boys) and parental involvement in PA projects of the CC accounted for 18% of the variance of children's skinfold thickness.

#### 3.2.2. Lasso Model

Lasso regression analysis was used to test robustness (accuracy and interpretability) of our results. Details are provided in Additional file 1. In summary, the standardized coefficient (SC) cutoff was, arbitrary, set at 0.07. Lasso results were very similar to the regression analysis for most of the correlates identified for TPA, MVPA, SB, BMI, and SF, corroborating the importance of these correlates mentioned in the current study. However, few discrepancies between regression and lasso were identified: child interaction with others in CC was associated with overall TPA and MVPA in the regression model but was not significant in the lasso model (SC = 0.06 and 0.03, respectively). Also, parental PA involvement in PA projects of the CC was associated with lower skinfold thickness in the regression model, but it was not significant in the lasso model (SC = −0.03). On the other side, some correlates were significant in the lasso model but not in the regression model: sex (boys), sociocultural region (German), higher SES of the CC, and larger CC surface were associated with less time spent in SB during CC attendance days (SC = 0.10, −0.10, 0.07, and −0.08, respectively). Additionally, sociocultural region (German) and larger CC surface were also shown to be associated with less time spent in overall SB in the lasso model (SC = 0.13 and 0.08, respectively). Finally, age was significant for skinfold thickness in the lasso model (SC = −0.07).

## 4. Discussion

Based on the socioecological model of health behavior, this study aimed at identifying a broad range of potential CC correlates of PA, SB, and adiposity in a large sample of preschool children using a cross-sectional design. Those correlates tested were selected based on the following 5 domains: demographic/biological, psychological/cognitive/emotional, behavioral, sociocultural, and physical environment [20]. Thus, by using a socioecological model and taking into account a larger spectrum of CC correlates, we could identify novel and mostly modifiable correlates of daily PA and SB during CC days and/or of overall daily PA, such as increased child-initiated interactions with other children during CC, mixing different ages within a CC group, the presence of a written PA policy, and a larger CC surface. Parental involvement in PA projects of the CC was a correlate of reduced body fat.

### 4.1. Daily Physical Activity during Childcare Attendance Days

We measured PA during CC attendance days (full-day attendance), which is comparable to most other studies that measured PA during CC. In line with previous studies, we found that sex (boys) and age (older children) were consistent correlates of increased PA in preschool children [7, 17, 23, 57–60]. This compelling evidence suggests that gender-specific strategies could be helpful when arranging the CC environment to promote PA. For example, providing more intentional opportunities for girls to initiate activities might increase their motivation and involvement in PA. Another potential strategy to increase young girls PA during CC might be to give more emphasis to team-oriented activities which consequently might increase girl's enjoyment of PA.

Using the socioecological model proposed by Sallis et al. [20], the present study identified three previously unmeasured CC correlates of preschoolers' PA and SB. These included child-initiated interactions with other children during CC, mixing different ages within a CC group, and the presence of a written PA policy. Indeed, children interacting with each other boosts child-initiated activities which have been associated with higher PA levels [11]. In line with the theory of observational learning, younger children may look up to older children and mimic their behaviors [61]. Therefore, grouping older and younger children may encourage younger children to engage more in PA behaviors if the older children show the desired behavior. Finally, having a written policy defining the objectives, means, or strategies to promote PA is essential in elevating the importance of PA in a CC. Such policy can provide a conceptual framework and institutional commitment that would support participation, enjoyment, and safety in PA for preschoolers [62]. Interestingly, in the current sociocultural and physical CC environments in Switzerland, generic “activities” such as promoting child interactions and mixing ages within a CC group were stronger correlates of PA and SB than more “traditional” and PA-specific correlates. Based on these results, we would encourage spontaneous interaction between children of different age groups, in accordance with a previous study from the UK [16]. For example, encouraging free play activities where children cheer, imitate, and cooperate with each other will potentially boost their PA behaviors.

Contrary to findings in the current study, previous studies, mostly performed in the US, have found correlates such as staff training and education, structured PA, time devoted to indoor and outdoor PA opportunities, the presence of indoor and outdoor play spaces, portable equipment, and outdoor fixed equipment were associated with PA during CC attendance [7, 9, 10, 14, 16, 19, 63]. In our study, mobile equipment was borderline significant. The abovementioned differences could be attributable to study methodologies (choice of correlates, categorization and conceptualization of domains, and choice of observation instrument used to capture CC correlates), data acquisition of PA measures, and/or different sociocultural and political environments between countries.

### 4.2. Overall Daily Physical Activity (Childcare and Nonchildcare Days)

Previous studies looking at CC correlates focused on PA during CC attendance. In addition to this, we were also interested in investigating the CC correlates that are related to overall PA including all days, as it is this overall PA that is important for overall health outcomes [33]. The current study found that child-initiated interactions with other children during CC, mixing different ages within a CC group, and the presence of a larger CC surface were related to a higher overall daily PA and/or more time spent in overall MVPA (CC and non-CC days). Additionally, parental involvement in PA projects of the CC was related to higher overall daily SB. The latter could hint toward a compensatory response behavior outside the CC environment. To our knowledge, only one previous study investigated the effects of the CC characteristics on PA over the 24-hour day during one day of observation [32]. In contrast to our results, the authors found that time provided outdoor and total active time were associated with higher MVPA. Similar to our data, they did not find that staff PA training, presence of indoor play space, and portable or fixed play equipment were correlates of children's MVPA. Thus, strategies in the CC promoting positive PA behaviors do not seem to lead to a complete compensation outside of the CC by reducing PA at home and might potentially even transfer outside the CC environment.

#### 4.2.1. Adiposity

It was previously shown that obesogenic features of the CC environment have a substantial influence on obesity development in young children [19]. In our study, we found that age (older) was associated with lower BMI. We also found that sex (boys) and parental involvement in PA projects of the CC were associated with reduced body fat. Because boys are more engaged in higher PA intensities than girls, these specific intensities can offer protection against the development of obesity. Indeed, vigorous PA is strongly associated with lower adiposity in preschoolers [64].

The importance of parental involvement to change children's lifestyle has been reported in previous successful family-based interventions [65, 66]. In our study, parental involvement in PA projects of the CC was associated with more overall SB and lower body fat. The reduced overall SB might be a compensatory mechanism, but it is not clearly understood and should be investigated in other studies. Regarding body fat, it could suggest that parental involvement might indirectly lead to favourable body composition since PA has a direct relationship with body fat and health behaviors [67]. On the other side, parental involvement in PA projects of the CC could just be a marker of increased overall parental commitment. In contrast to existing CC intervention studies, we only know of one study, done in the US, that looked at several CC correlates of adiposity [19]. The authors showed that less opportunities for sedentary activities, less minutes spent in SB, more time spent in active play, and less offers of high fat/high sugar foods in CC were all associated with lower BMI or lower risk of being overweight. Interestingly, they showed that 75% of the children used the computer on their observation day, while in the current study, no computer was used by the children in the CCs (data not shown). Thus, large sociocultural discrepancies between countries may impact on differences in the results. The observed differences between the current study and the previous one could also be attributable to the choice in the observation instrument for the correlates and in the PA measurements (wristwatch). Nonetheless, offering children opportunities to be active and involving parents in PA projects might encourage PA participation and/or protect against adiposity. For instance, informing parents about in-care PA behaviors of their child would improve communication between staff and parents, and eventually encourage parents to engage in the PA projects the CC.

Strengths of the current study include the conceptualization of potential correlates of PA, SB, and adiposity across 5 domains of the socioecological model of health behavior. Other strengths comprise the inclusion of novel correlates, multimethod approach including direct measurements, educator and parent-report information, objectively measured PA and SB both on CC attendance days and during CC and non-CC stay, and the relatively large study sample. Also, analyses applied of two statistical models, one of them novel, showed very similar results and gave a better picture on the importance of putative correlates and the robustness of our results. Limitations include the cross-sectional design which limits causation. Also, we did not perform a direct observation of the CC environment. However, the main responsible educator of the CC group shared the information regarding the CC correlates over several weeks of observation as opposed to an observation that lasts usually one day. This might especially be important for correlates such as daily PA and time spent outside that might fluctuate between days and according to the weather and season. The high number of correlates tested might have caused random findings. However, the lasso model through the penalized regression limited irrelevant correlates to be included in the model. Physical activity measurement during days with full-day CC attendance was not exactly time stamped to capture PA exactly during CC time only, which may in turn resulted in misclassification PA during CC. Also, two items from the psychological/cognitive/emotional domain (“playing peers” and “staff support”) that were developed to meet the needs of our study were not validated in young children.

## 5. Conclusions

In conclusion, our findings provide evidence for novel CC correlates of preschoolers PA, SB, and adiposity using a socioecological model. Enabling spontaneous PA interactions between children of different age groups, establishing clear written PA policies, and a larger collaboration with parents in PA projects of the CC might lead to increased PA and reduced body fat in children. Integrating these mostly modifiable factors might be beneficial when planning future interventions.

## Figures and Tables

**Table 1:** Potential childcare correlates of physical activity, sedentary behavior, and adiposity.

Correlates	Source	Description	Mean (SD) or %
(1) Demographic-biological			
Age (years)	Parental report	Child's age in years	3.9 (0.7)
Sex (% male)	Parental report	Child's sex	53
Sociocultural region (% German)	Regional location of the CC	German or French speaking region of Switzerland	74
CC SES (% high)	Swiss neighbourhood index	Socioeconomic status of the community of the CC	67
CC rural/urban (% urban)	Location of the CC	Urban defined as biggest cities of each canton and cities > 100,000 inhabitants	60

(2) Psychological-cognitive-emotional			
Staff support	Direct observation by research staff	Staff support for children in finding solutions to problems: Scale 1–5 increasing with more support	4.2 (0.7)
Child-initiated interaction	SDQ questionnaire *Subscale: peer relationship problems*	Child goes toward other children: Likert's scale 0–4 increases with child going more toward other children	3.2 (0.8)
Playing peers	CC questionnaire	*N* of peers the child plays with during CC	4.5 (3.9)

(3) Behavioral			
*Eating behavior*			
Interest in food^*∗*^	CEBQ questionnaire Subscale: enjoyment of food	Child's interest in food: Likert's scale 0–4 increases with child's interest in food	3.0 (0.8)
Eating when upset^*∗*^	CEBQ questionnaire Subscale: emotional overeating	Child eating more when upset: Likert's scale 0–4 increases with child eating more when upset	0.2 (0.4)
Leaving food on the plate^*∗*^	CEBQ questionnaire Subscale: satiety responsiveness	Child leaving food on the plate after meals: Likert's scale 0–4 increases with child leaving food on plate	1.7 (0.9)

(4) Sociocultural			
*Organisational policies*			
Mixing different ages	CC questionnaire	*N* of different age classes within a CC group	4.2 (1.3)
*PA policies*			
Staff participation in PA	NAP SACC questionnaire Subscale: PA	Staff participation during free PA: Likert's scale 0–3 increases with more staff participation	2.0 (0.7)
Staff PA training	NAP SACC questionnaire Subscale: PA	*N* of staff members with PA training and education	0.6 (1.1)
Written PA policy (% yes)	NAP SACC questionnaire Subscale: PA	Presence of a written policy for promoting PA	68
Daily PA (%)	NAP SACC questionnaire Subscale: PA	Time dedicated to daily PA per CC	
		<30 minutes	2
		31–60 minutes	25
		61–90 minutes	20
		> 90 minutes	53
Daily structured PA (min)	NAP SACC questionnaire Subscale: PA	Time in minutes dedicated to daily structured PA	30.8 (25.1)
Parental PA involvement (% yes)	CC questionnaire	Parental involvement in PA projects set up by the CC	48
*Nutritional policies*			
Staff nutrition training^*∗*^	NAP SACC questionnaire Subscale: nutrition	*N* of staff members with children nutritional training and education	1.5 (0.9)
Staff food encouragement^*∗*^	NAP SACC questionnaire Subscale: nutrition	Staff encouraging children to health all kind of healthy foods: Likert's scale 1–4 increases with more encouragement from staff	3.8 (0.5)
Children self-service^*∗*^	NAP SACC questionnaire Subscale: nutrition	Children serve themselves without any help from staff: Likert's scale 1–4 increases with more food self-service from children	3.1 (0.9)
Clean plate^*∗*^	NAP SACC questionnaire Subscale: nutrition	Children are encouraged to finish their plate: Likert's scale 1–4 increases with more encouragement from staff to finish their plate	1.7 (1.0)
Fruits availability^*∗*^	NAP SACC questionnaire Subscale: nutrition	Frequency of fruits availability in CC: Likert's scale 1–4 increases with more fruits availability	3.8 (0.4)
Vegetables availability^*∗*^	NAP SACC questionnaire Subscale: nutrition	Frequency of vegetables availability in CC: Likert's scale 1–4 increases with more vegetables availability	3.4 (0.5)
Sweet drinks availability^*∗*^	NAP SACC questionnaire Subscale: nutrition	Frequency of sweet drinks availability in CC: Likert's scale 0–3 increases with more sweets drinks availability	0.1 (0.4)
Juices availability^*∗*^	NAP SACC questionnaire Subscale: nutrition	Frequency of juices availability in CC: Likert's scale 0–3 increases with more juices availability	0.7 (1.1)
Water availability^*∗*^	NAP SACC questionnaire Subscale: Nutrition	Availability of drinking water in CC: Likert's scale 0–3 increases with more water being freely available	2.9 (0.4)
Using food as reward^*∗*^	NAP SACC questionnaire Subscale: nutrition	Food is used to reward desired behavior: Likert's scale 0–3 increases with more food reward	0.1 (0.3)
Using food as regulator^*∗*^	NAP SACC questionnaire Subscale: nutrition	Food is used to control behavior or as punishment: Likert's scale 0–3 increases with more often food used to control behavior	0.1 (0.3)

(5) Physical environment			
CC surface	CC questionnaire	Ratio of the CC surface (m^2^) per number of children	7.0 (5.1)
CC attendance	Parental report	*N* of full days child attending CC per week	2.8 (1.2)
*PA*			
PA indoor space (% yes)	NAP SACC questionnaire Subscale: PA	Presence of indoor space dedicated for PA	80
PA outdoor space (% yes)	NAP SACC questionnaire Subscale: PA	Presence of outdoor space dedicated for PA	91
Fixed PA equipment	NAP SACC questionnaire Subscale: PA	*N* of fixed equipment related to PA	2.9 (2.5)
Mobile PA equipment	NAP SACC questionnaire Subscale: PA	N of mobile equipment related to PA	18.9 (19.8)

*N* = number; CC = childcare; SES = socioeconomic status; SDQ = strength and difficulties questionnaire; CEBQ = child eating behavior questionnaire; PA = physical activity; NAP SACC = nutrition and physical activity self-assessment for childcare; ^*∗*^ = correlates included in the models with adiposity as outcome. We used a modified version of the NAP SACC questionnaire.

**Table 2:** Descriptive characteristics of the participants.

Characteristics	Total population (*n*=476)
Age (years)	3.9 ± 0.7
Sex	
Boys, *n* (%)	246 (53)
Girls, *n* (%)	217 (47)
Sociocultural region	
German speaking, *n* (%)	342 (74)
French speaking, *n* (%)	121 (26)
Full-day attendance at CC	2.8 (1.2)

*Adiposity*	
Body mass index (kg/m^2^)	16.02 ± 1.35
Skinfold thickness (mm)	25.93 ± 5.53
Weight status^*∗*^	
Overweight, *n* (%)	82 (18)
Obese, *n* (%)	24 (5)

*Daily physical activity during CC attendance days*	
TPA (cpm)	624 ± 166
MVPA (min/d)	46 ± 58
SB (min/d)	357 ± 65

*Overall daily physical activity (CC and non-CC days)*	
TPA (cpm)	622 ± 153
MVPA (min/d)	46 ± 23
SB (min/d)	366 ± 56

CC = childcare; TPA = total physical activity; LPA = light physical activity; MPA = moderate physical activity; MVPA = moderate-to-vigorous physical activity; VPA = vigorous physical activity; SB = sedentary behavior; cpm = counts per minute; min/d = minutes per day. Daily physical activity during CC attendance days denotes the physical activity and days where the child attends CC during the full day. Overall daily physical activity denotes physical activity during all days, i.e., CC and non-CC days. ^*∗*^Based on the WHO criteria [40]. Cutoff for the physical activity intensities are based on Butte [38].

**Table 3:** Associations of childcare correlates with physical activity and sedentary behavior.

Outcomes	Correlates	*β*-coefficient	95% CI	*p* value
*(a) Daily PA during childcare attendance days (full-day attendance)*				
Total PA (cpm)	Age (years)	45.14	(19.35, 70.94)	**0.001**
	Sex	−52.96	(−84.65, −21.26)	**0.001**
	Child-initiated interaction^*∗*^	27.68	(4.67, 50.69)	**0.02**
	Mixing ages within a CC group	26.61	(7.02, 46.21)	**0.01**
	Written PA policy	53.28	(12.58, 93.98)	**0.01**
Moderate and vigorous PA (min/d)	Age (years)	10.09	(6.28, 13.90)	**0.001**
	Sex	−10.00	(−14.90, −5.11)	**0.001**
	Child-initiated interaction^*∗*^	4.45	(1.11, 7.79)	**0.01**
	Mixing ages within a CC group	3.74	(0.80, 6.67)	**0.01**
	Written PA policy	7.14	(1.40, 12.88)	**0.01**
Sedentary time (min/d)	Child-initiated interaction^*∗*^	−10.01	(−19.68, −0.33)	**0.043**
	Mixing ages within a CC group	−10.35	(−18.09, −2.61)	**0.01**
	Written PA policy	−23.92	(−39.99, −7.86)	**0.004**

*(b) Overall daily PA (childcare and nonchildcare days)*				
Total PA (cpm)	Age (years)	54.76	(33.23, 76.29)	**0.001**
	Sex	−58.97	(−86.64, −31.29)	**0.001**
	Child-initiated interaction^*∗*^	20.81	(0.79, 40.84)	**0.04**
	Mixing ages within a CC group	18.45	(1.07, 35.84)	**0.04**
Moderate and vigorous PA (min/d)	Age (years)	10.33	(7.24, 13.42)	**0.001**
	Sex	−11.39	(−15.41, −7.37)	**0.001**
	Child-initiated interaction^*∗*^	3.55	(0.65, 6.46)	**0.02**
	Mixing ages within a CC group	3.25	(0.71, 5.78)	**0.01**
	CC surface	0.51	(0.01, 1.01)	**0.047**
Sedentary time (min/d)	Sex	10.83	(0.24, 21.41)	**0.045**
	Child-initiated interaction^*∗*^	−8.95	(−16.76, −1.13)	**0.03**
	Parental PA involvement	14.66	(1.61, 27.71)	**0.03**

PA = physical activity; CI = confidence interval; CC = childcare; cpm = counts per minutes; min/d = minutes per day; ^*∗*^5-point Likert scale; *Sex* defines girls vs boys. *Child interaction* indicates child's tendency to go towards other peers while in CC. *Mixing ages* refers to the number of age classes in one group. *Written PA policy* refers to having a written PA policy promoting PA participation within the CC. *Mobile equipment* is expressed per 10 more equipment such as balls and frisbees. All outcome and predictor variables are unstandardized. The model always included age and sex. Daily physical activity during CC attendance days denotes the physical activity and days where the child attends CC during the full day. Overall daily physical activity denotes physical activity during all days, i.e., CC and non-CC days.

**Table 4:** Associations of childcare correlates with adiposity.

Outcomes	Correlates	*β*-coefficient	95% CI	*p* value
BMI	Age (years)	−0.32	(−0.53, −0.11)	**0.002**
Skinfold thickness	Sex	2.87	(1.81, 3.94)	**0.001**
	Parental PA involvement	−1.64	(−3.01, −0.28)	**0.02**

PA = physical activity; CI = confidence interval; CC = childcare; BMI = body mass index. *Sex* defines girls vs boys.

## Data Availability

The dataset supporting the conclusions of this article is available upon reasonable request to the corresponding author.

## Supplementary Materials

Supplementary Materials Table 5: associations of childcare correlates with physical activity and sedentary behavior using lasso. Table 6: associations of childcare correlates with body composition using lasso.
