# Supplementary material for: Childcare Correlates of Physical Activity, Sedentary Behavior, and Adiposity in Preschool Children: A Cross-Sectional Analysis of the SPLASHY Study
Source: J Environ Public Health. 2018 Nov 11;2018:9157194. doi: 10.1155/2018/9157194 (PMC6311763; doi:10.1155/2018/9157194)
Supplement: Supplementary Materials — Table 5: associations of childcare correlates with physical activity and sedentary behavior using lasso. Table 6: associations of childcare correlates with body composition using lasso. [file 9157194.f1.pdf]

Table 5. Associations of childcare correlates with physical activity and sedentary behavior using Lasso.

a) PA during childcare days (full-day attendance)

| Outcome | Predictors | Mean-lasso coefficient | SD | Number of selection |
|---|---|---|---|---|
| Total PA | **Age*** | **0.20** | **0.02** | **50** |
| | **Sex*** | **-0.15** | **0.02** | **50** |
| | **Mixing ages within a CC group*** | **0.13** | **0.05** | **50** |
| | **Written PA policy*** | **0.11** | **0.03** | **50** |
| | **Child-initiated interaction*** | **0.10** | **0.04** | **50** |
| | PA indoor space | 0.04 | 0.02 | 47 |
| | Parental PA involvement | -0.04 | 0.03 | 47 |
| | Staff PA training | -0.04 | 0.03 | 46 |
| | CC attendance | -0.04 | 0.03 | 46 |
| | CC surface | 0.04 | 0.03 | 38 |
| | Sociocultural region | 0.03 | 0.04 | 37 |
| | Daily Structured PA | -0.03 | 0.03 | 32 |
| | Playing peers | 0.03 | 0.03 | 31 |
| | PA outdoor space | -0.02 | 0.02 | 33 |
| | Staff support | -0.02 | 0.03 | 29 |
| | Daily PA | -0.02 | 0.03 | 22 |
| | Mobile PA equipment | 0.02 | 0.03 | 16 |
| | CC SES | -0.01 | 0.02 | 29 |
| | Fixed PA equipment | -0.01 | 0.02 | 28 |
| | CC rural urban | 0.01 | 0.02 | 28 |
| | Staff PA participation | 0.01 | 0.02 | 21 |
| Moderate-and-vigorous PA | **Age*** | **0.28** | **0.02** | **50** |
| | **Sex*** | **-0.18** | **0.02** | **50** |
| | **Mixing ages within a CC group*** | **0.11** | **0.05** | **49** |
| | **Child-initiated interaction*** | **0.10** | **0.04** | **49** |
| | **Written PA policy*** | **0.08** | **0.03** | **49** |
| | Staff PA training | -0.03 | 0.02 | 38 |
| | PA indoor space | 0.03 | 0.03 | 38 |
| | CC surface | 0.03 | 0.03 | 36 |
| | Fixed PA equipment | -0.03 | 0.03 | 36 |
| | Daily structured PA | -0.03 | 0.03 | 33 |
| | Playing peers | 0.03 | 0.04 | 29 |
| | Daily PA | -0.03 | 0.03 | 28 |
| | Mobile PA equipment | 0.02 | 0.04 | 17 |
| | Parental PA involvement | -0.01 | 0.02 | 25 |
| | CC attendance | -0.01 | 0.02 | 25 |
| | PA outdoor space | -0.01 | 0.02 | 22 |
| | Staff PA participation | 0.01 | 0.02 | 21 |
| | Sociocultural region | 0.01 | 0.01 | 19 |
| | CC rural urban | 0.01 | 0.02 | 19 |
| | CC SES | 0.01 | 0.02 | 15 |
| | Staff support | 0.00 | 0.01 | 11 |
| Sedentary time | **Mixing ages within a CC group*** | **-0.14** | **0.05** | **50** |
| | **Written PA policy*** | **-0.14** | **0.04** | **50** |
| | Parental PA involvement | 0.10 | 0.03 | 49 |
| | **Child-initiated interaction*** | **-0.09** | **0.04** | **50** |
| | Sex | 0.09 | 0.02 | 50 |
| | CC surface | -0.06 | 0.03 | 46 |
| | Sociocultural region | -0.06 | 0.04 | 45 |
| | Staff PA training | 0.05 | 0.03 | 46 |
| | CC attendance | 0.05 | 0.04 | 45 |
| | CC SES | 0.04 | 0.03 | 45 |
| | Staff support | 0.04 | 0.04 | 38 |
| | PA indoor space | -0.03 | 0.02 | 40 |
| | CC rural urban | -0.03 | 0.03 | 38 |
| | Daily structured PA | 0.03 | 0.03 | 34 |

| | | | | |
|---|---|---|---|---|
| | Age | -0.02 | 0.02 | 50 |
| | PA outdoor space | 0.02 | 0.02 | 37 |
| | Playing peers | -0.02 | 0.03 | 26 |
| | Daily PA | 0.02 | 0.03 | 22 |
| | Mobile PA equipment | -0.01 | 0.02 | 18 |
| | Fixed PA equipment | 0.00 | 0.02 | 24 |
| | Staff PA participation | 0.00 | 0.01 | 12 |

PA= physical activity; SD= standard error; CC= childcare.
* Significant in the regression model
All outcomes and predictors are standardized. The model always included age and sex.

b) Overall PA (all days, both home and childcare days)

| Outcome | Predictors | Mean-lasso coefficient | SD | Number of selection |
|---|---|---|---|---|
| Total PA | **Age*** | **0.27** | **0.01** | **50** |
| | **Sex*** | **-0.18** | **0.01** | **50** |
| | **Mixing ages within a CC group*** | **0.09** | **0.03** | **50** |
| | CC surface | 0.06 | 0.03 | 49 |
| | Staff PA training | -0.06 | 0.03 | 49 |
| | **Child-initiated interaction *** | **0.06** | **0.03** | **46** |
| | CC attendance | -0.05 | 0.04 | 43 |
| | Parental PA involvement | -0.04 | 0.02 | 49 |
| | Sociocultural region | 0.02 | 0.02 | 35 |
| | CC rural urban | 0.02 | 0.02 | 34 |
| | Written PA policy | 0.02 | 0.02 | 32 |
| | Staff PA participation | 0.01 | 0.02 | 21 |
| | PA indoor space | 0.01 | 0.02 | 18 |
| | Fixed PA equipment | 0.00 | 0.01 | 13 |
| | Mobile PA equipment | 0.00 | 0.02 | 10 |
| | CC SES | 0.00 | 0.01 | 9 |
| | Playing peers | 0.00 | 0.01 | 8 |
| | Staff support | 0.00 | 0.01 | 6 |
| | Daily PA | 0.00 | 0.01 | 6 |
| | PA outdoor space | 0.00 | 0.00 | 6 |
| | Daily structured PA | 0.00 | 0.01 | 5 |
| Moderate-and-vigorous PA | **Age*** | **0.32** | **0.02** | **50** |
| | **Sex*** | **-0.24** | **0.01** | **50** |
| | **Mixing ages within a CC group*** | **0.12** | **0.04** | **50** |
| | **Child-initiated interaction*** | **0.09** | **0.03** | **50** |
| | **CC surface*** | **0.07** | **0.03** | **50** |
| | Staff PA training | -0.06 | 0.02 | 49 |
| | CC rural urban | 0.05 | 0.02 | 46 |
| | Fixed PA equipment | -0.04 | 0.03 | 46 |
| | CC SES | 0.04 | 0.03 | 44 |
| | Playing peers | 0.04 | 0.03 | 38 |
| | Mobile PA equipment | 0.04 | 0.04 | 30 |
| | Parental PA involvement | -0.03 | 0.02 | 43 |
| | Written PA policy | 0.03 | 0.02 | 43 |
| | CC attendance | -0.03 | 0.03 | 35 |
| | Staff PA participation | 0.01 | 0.02 | 26 |
| | Daily PA | -0.01 | 0.02 | 24 |
| | Daily structured PA | -0.01 | 0.02 | 24 |
| | PA outdoor space | 0.00 | 0.01 | 20 |
| | Staff support | 0.00 | 0.01 | 15 |
| | PA indoor space | 0.00 | 0.01 | 16 |
| | Sociocultural region | 0.00 | 0.02 | 5 |
| Sedentary time | Sociocultural region | -0.11 | 0.03 | 50 |
| | **Parental PA involvement*** | **0.11** | **0.02** | **50** |
| | **Sex*** | **0.09** | **0.01** | **50** |
| | **Child-initiated interaction*** | **-0.09** | **0.03** | **49** |
| | CC surface | -0.07 | 0.03 | 48 |
| | CC rural urban | -0.06 | 0.03 | 49 |

| | | | | |
|---|---|---|---|---|
| | Written PA policy | -0.06 | 0.03 | 48 |
| | CC attendance | 0.05 | 0.04 | 44 |
| | Staff PA training | 0.04 | 0.02 | 46 |
| | Mixing ages within a CC group | -0.04 | 0.03 | 44 |
| | Age | -0.02 | 0.01 | 50 |
| | PA indoor space | -0.02 | 0.02 | 35 |
| | Mobile PA equipment | 0.01 | 0.01 | 18 |
| | CC SES | 0.00 | 0.00 | 7 |
| | Daily PA | 0.00 | 0.01 | 7 |
| | Playing peers | 0.00 | 0.01 | 11 |
| | Staff PA participation | 0.00 | 0.01 | 13 |
| | Fixed PA equipment | 0.00 | 0.01 | 9 |
| | Staff support | 0.00 | 0.00 | 5 |
| | Daily structured PA | 0.00 | 0.01 | 11 |
| | PA outdoor space | 0.00 | 0.00 | 6 |

PA= physical activity; SD= standard error; CC= childcare.
* Significant in the regression model.
All outcomes and predictors are standardized. The model always included age and sex.

Table 6. Associations of childcare correlates with body composition using Lasso.

| Outcome | Predictors | Mean-lasso coefficient | SD | Number of selection |
|---|---|---|---|---|
| BMI | **Age*** | **-0.15** | **0.01** | **50** |
| | Sociocultural region | 0.03 | 0.02 | 47 |
| | Sex | -0.02 | 0.01 | 50 |
| | Mixing ages within a CC group | 0.01 | 0.02 | 29 |
| | Leaving food on the plate | -0.01 | 0.02 | 15 |
| | Staff food encouragement | 0.01 | 0.01 | 8 |
| | CC surface | 0.00 | 0.01 | 14 |
| | Clean plate | 0.00 | 0.01 | 10 |
| | Daily structured PA | 0.00 | 0.01 | 8 |
| | Staff nutrition training | 0.00 | 0.01 | 7 |
| | Playing peers | 0.00 | 0.01 | 6 |
| | Using food as reward | 0.00 | 0.01 | 4 |
| | Staff support | 0.00 | 0.00 | 3 |
| | Juices availability | 0.00 | 0.01 | 3 |
| | Water availability | 0.00 | 0.00 | 2 |
| | Interest in food | 0.00 | 0.00 | 2 |
| | Sweet drinks availability | 0.00 | 0.00 | 2 |
| | Eating when upset | 0.00 | 0.00 | 1 |
| | Children self service | 0.00 | 0.00 | 1 |
| | CC attendance | 0.00 | 0.00 | 1 |
| | Vegetables availability | 0.00 | 0.00 | 1 |
| | Written PA policy | 0.00 | 0.00 | 1 |
| | PA indoor space | 0.00 | 0.00 | 1 |
| | Fruits availability | 0.00 | 0.00 | 0 |
| | Mobile equipment | 0.00 | 0.00 | 0 |
| | Using food as regulator | 0.00 | 0.00 | 0 |
| | CC SES | 0.00 | 0.00 | 0 |
| | CC rural urban | 0.00 | 0.00 | 0 |
| | Staff PA training | 0.00 | 0.00 | 0 |
| | PA outdoor space | 0.00 | 0.00 | 0 |
| | Child-initiated interaction | 0.00 | 0.00 | 0 |
| | Daily PA | 0.00 | 0.00 | 0 |
| | Staff PA participation | 0.00 | 0.00 | 0 |
| | Fixed PA equipment | 0.00 | 0.00 | 0 |
| | Parents PA involvement | 0.00 | 0.00 | 0 |
| Skinfold thickness | **Sex*** | **0.26** | **0.01** | **50** |
| | Age | -0.07 | 0.01 | 50 |
| | Staff support | -0.05 | 0.03 | 50 |
| | CC rural urban | -0.05 | 0.03 | 48 |

| | | | | |
|---|---|---|---|---|
| | **Parental PA involvement*** | **-0.03** | **0.04** | **35** |
| | CC SES | 0.02 | 0.02 | 32 |
| | Written PA policy | 0.02 | 0.03 | 26 |
| | Staff PA training | 0.02 | 0.03 | 26 |
| | Vegetables availability | -0.01 | 0.02 | 26 |
| | Staff nutrition training | 0.01 | 0.01 | 21 |
| | Staff PA participation | -0.01 | 0.02 | 19 |
| | CC attendance | 0.01 | 0.01 | 16 |
| | Mobile equipment | -0.01 | 0.02 | 14 |
| | Sociocultural region | -0.01 | 0.02 | 13 |
| | Staff food encouragement | 0.01 | 0.02 | 12 |
| | Juices availability | -0.01 | 0.02 | 8 |
| | Children self service | 0.00 | 0.01 | 15 |
| | PA outdoor space | 0.00 | 0.01 | 12 |
| | Child-initiated interaction | 0.00 | 0.01 | 10 |
| | Eating when upset | 0.00 | 0.01 | 9 |
| | PA indoor space | 0.00 | 0.01 | 9 |
| | Leaving food on the plate | 0.00 | 0.01 | 9 |
| | Using food as regulator | 0.00 | 0.01 | 9 |
| | CC surface | 0.00 | 0.01 | 5 |
| | Fixed PA equipment | 0.00 | 0.01 | 5 |
| | Water availability | 0.00 | 0.01 | 4 |
| | Sweet drinks availability | 0.00 | 0.01 | 4 |
| | Fruits availability | 0.00 | 0.00 | 4 |
| | Using food as reward | 0.00 | 0.01 | 3 |
| | Interest in food | 0.00 | 0.01 | 3 |
| | Clean plate | 0.00 | 0.01 | 3 |
| | Playing peers | 0.00 | 0.00 | 2 |
| | Mixing ages within a CC group | 0.00 | 0.00 | 1 |
| | Daily structured PA | 0.00 | 0.00 | 1 |
| | Daily PA | 0.00 | 0.00 | 1 |

PA= physical activity; SD= standard error; CC= childcare.
* Significant in the regression model.
All outcomes and predictors are standardized. The model always included age and sex.
